# A Critical Consideration of Two Methodologies of General Jurisprudence

**DOI:** 10.1093/ojls/gqaf020

**Published:** 2025-06-05

**Authors:** Jorge Cortés-Monroy

**Keywords:** general jurisprudence, conceptual analysis, armchair theorising, naturalised jurisprudence, legal theory

## Abstract

Whether in the form of conceptual analysis or as grounding reduction, armchair theorising has been the main method of theory construction in general jurisprudence in the English-speaking world. Given important deficiencies in this way of proceeding, empiricist jurisprudence, particularly in the form of reductive naturalism, has emerged as an alternative to armchair theorising and has gained some support within the discipline. In this article, I argue that both of these methodological positions ultimately provide us with partial and thus inadequate explanations of the *complex* social phenomenon of law. In their failure, however, each position does get something right. I then further argue that to make good of these positions’ strengths and simultaneously correct their shortcomings, we need to be able to navigate between the self-understanding of participants of the social practice of law and the viewpoints of social scientists and critical observers.

## 1. Introduction

Either in the form of conceptual analysis or as grounding reduction, armchair theorising has been almost the exclusive method for theory construction in general jurisprudence in the English-speaking world. Given substantial deficiencies in this way of proceeding, empiricist jurisprudence, particularly in the form of a reductive naturalism, has emerged as an alternative and gained some support within the discipline (most notably, within US law schools). In this article, I argue that both methodological positions—which are, in fact, the two main existing methodological approaches to general jurisprudence—fail to provide us with adequate explanations of the *complex* social phenomenon of law.

In their failure, however, each position does get something right. Armchair jurisprudence is right that legal theory *can* say something of *general* interest in understanding the social phenomenon of law (thus counterweighting the localising tendencies of the empirical social sciences) and that, as at least some forms of conceptual analysis maintain, an adequate explanation of the general features of law requires accounting for the self-understanding of the practice. And radical naturalism is right that an adequate explanation of such a phenomenon requires considering it from an observational point of view. I will thus argue that what we need is a method that enables us to *legitimately* navigate between these two dimensions. Such a method is, in fact, a reconstructive one in which the theorist works from four ends: the general propositions that he formulates; the judgments that participants of the practice intuitively make; the best empirical observations of the social phenomenon at stake (paradigmatically provided by the social sciences); and the perspective of other social practices external to law. Only this way, I will conclude, will we be able to give an adequate explanation of law as a *complex* social phenomenon.

The article proceeds in the following way. Section 2 reconstructs and criticises two forms of armchair jurisprudence: conceptual analysis and metaphysical reduction. Importantly, conceptual analysis is not a univocal enterprise, but it has been practised in different ways with different degrees of explanatory ambitions. All these projects, however, share the methodological feature with grounding reductions that, in formulating his inferences, the theorist works *exclusively* from his *own* intuitions. Considering the different ways in which this inquiry has been carried out will provide us with resources to formulate the methodological challenge that we have in front of us.

Section 3 deals with the main way in which naturalism has been conceived in legal theory: as an empiricist, reductively naturalistic jurisprudence. It also puts forth a pragmatic attempt to vindicate normative and hermeneutic explanations that, if successful, will allow us to see the shortcomings of such a reductively naturalised jurisprudence.

Finally, section 4 formulates, as clearly as I can, the methodological challenge that we have in front of us, given the strengths and weaknesses of both former positions. This, however, will lead us to the question of whether it is legitimate and necessary for one and the same discipline to be concerned with all the different perspectives from which the phenomenon of law can be studied. Needless to say, we can only recommend the kind of methodological dualism that I will propose here if we can answer this question in the affirmative.

With the roadmap set out, I will begin with armchair jurisprudence.

## 2. Armchair Jurisprudence

For our purposes, armchair theorising is that form of theory construction that uses the theorist’s *own* intuitions as the *exclusive* evidential source for the formulation of theoretical propositions about the phenomenon at stake—in our case, about the social phenomenon of law.^1^ When theorising from the armchair, then, the theorist appeals exclusively to his own intuitions to formulate the premises for his inferences.^2^ Thus, I include conceptual analysis in jurisprudence as well as ‘grounding reductive’ theories of law as forms of armchair jurisprudence. As we will see, what they both have in common is that they assume that further experience in order to broaden the pool of intuitive judgments available to us or find new evidential sources of a different kind is unnecessary.^3^

Let us look at each one of these in turn.

### A. Conceptual Jurisprudence

As a method of philosophical inquiry, conceptual analysis is concerned with ascertaining the meaning of a concept by determining the properties in virtue of which the concept applies to a certain phenomenon-referent.^4^ After the linguistic turn in philosophy in the 20th century, talk of ‘essential properties’ shifted from those features that things themselves were supposed to possess (and that made them what they were) to the features that their concepts singled out. This ‘move’ enabled philosophers to avoid the trap of metaphysical essentialism when attempting to contribute to our knowledge of the world—now, instead of indulging in futile disquisitions about the possibility of knowing ‘things in themselves’, they just needed to analyse the meaning of their concepts.^5^ Thus, the idea that conceptual analysis was the right task for philosophy (and all that could be legitimately asked from it) was already dominant in the English-speaking world by the middle of the 20th century.

Whatever its merits, conceptual analysis has been the main method that general jurisprudence has adopted to investigate the phenomenon of law. This way, general jurisprudents have typically been occupied with ascertaining the meaning of the concept of law and other law-related concepts. It is important to note, however, that philosophers embarked on this task are usually eager to emphasise that their business is *not* that of offering lexical definitions.^6^ Rather, they have insisted that what they seek is to elucidate that part of the world to which the concept of law and other law-related concepts refer. In his response to Dworkin’s semantic sting argument, HLA Hart himself emphasised this point,^7^ and other analytical legal philosophers have done so as well.^8^ Thus, if we take these philosophers at their word, we must say that any insight that we gain into the concept-term ‘law’ or the fact that we develop it in new ways as the result of conceptual analysis must be just the by-product of our interest in the nature of the social phenomenon itself.

This, then, leaves us with two alternatives to make sense of the point of conceptual analysis in general jurisprudence. On the one hand, some conceptualists have sought to determine the meaning of the *concept* of ‘law’ and of other law-related *concepts* (not merely of the words that designate them) by identifying the properties that constitute something as falling within their reference (ie properties without which such a thing would not be law or that other law-related phenomenon under consideration^9^). This kind of inquiry is commonly known as conceptual analysis in its ‘modest’ mode.^10^ According to this less ambitious mode, conceptual analysis attempts to bring legal phenomena into a clearer focus without trying to say anything about its actual character—this latter task should be left to empirical investigation.

Because it is not mere linguistic analysis, even in its modest mode, *conceptual* analysis must involve *more* than (i) determining the meaning of words or expressions and the criteria for their application by identifying shared intuitions about their use.^11^ This is, in fact, just the starting point. In order to determine the meaning of our *concepts* and thus ‘sharpen our perception of phenomena’, conceptual analysis *also* involves determining (ii) the deeper *commitments* entailed by the shared patterns of word usage that we have initially discovered and (iii) the general *principles* that explain those commitments.^12^ In short, ‘modest’ conceptual analysis aims at making explicit what is implicit in shared linguistic usage, and it does so without trying to say anything about the actual character of the phenomenon at stake.

But, as is well known, there is also a more ambitious mode of conceptual analysis, one that in legal philosophy has indeed sought to tell us something about the *actual* character of the social phenomenon of law—ie about how that part of the world that is *legal* actually works. This type of inquiry is commonly known as conceptual analysis in its ‘immodest’ mode,^13^ and it was referred to by HLA Hart as a kind of ‘descriptive sociology’.^14^ Philosophers have considered this kind of conceptual analysis to be legitimate particularly in the case of socially constructed phenomena—phenomena wholly constituted by language.^15^ Here, the analysis of concepts reveals the beliefs and attitudes which, by being held by a certain group of people, *constitute* the social phenomena in the first place.^16^ An ‘immodest’ kind of conceptual analysis thus becomes a metaphysical inquiry. By determining the beliefs and attitudes that constitute a socially constructed phenomenon through the analysis of its concept, such an inquiry seeks to tell us something about the nature or essence of law.

While most philosophers seem to consider this a legitimate way in which conceptual analysis could contribute to our understanding of what a certain part of the world is like, there is a second way in which ‘conceptual analysis’ could attempt to say something about the actual character of the social phenomenon of law beyond the beliefs and attitudes that constitute it: through experientially informed speculation and direct empirical observation.^17^ The activity of the philosopher thus extended (when compared to mere linguistic analysis or even a ‘modest’ form of conceptual analysis) entails that it will frequently be the case that the claims derived from conceptual analysis appeal to intuitions of a different kind than those springing from linguistic competence.

In this, its utmost ambitious (or immodest) mode, conceptual jurisprudence would involve: (i) determining the meaning of words or expressions, and the criteria for their application; (ii) determining the deeper commitments that these patterns of word usage entail; (iii) determining the general principles that explain those commitments; (iv) experientially informed speculation about the properties of the things of which the concepts are concepts; and (v) direct empirical observation. In each of these five activities, the theorist will be working exclusively from his own intuitions (which he sometimes will take to be also the intuitions of the participants of the practice) and personal observations.

It is obvious that the more ambitious the aims of conceptual analysis are, the more controversial its legitimacy also is. Thus, given that the nature of our inquiry requires us to maximise our explanatory ambitions while preserving the legitimacy of our methods, either a modest mode of conceptual analysis or an immodest mode that tells us only about the beliefs and attitudes that constitute law as a social phenomenon will usually be considered the right form of inquiry. As we will see, however, conceptual analysis does not exhaust our alternatives, and it is precisely the goal of maximising our explanatory ambitions (without sacrificing the legitimacy of our methods) that requires us to proceed differently.

After Hart, most philosophers doing general jurisprudence in the English-speaking world have embraced the view that conceptual analysis is, in one mode or another, the best method to investigate the social phenomenon of law.^18^ Moreover, this conviction was reinforced by the fact that Joseph Raz, one of Hart’s most brilliant students, also practised a form of conceptual jurisprudence. But can conceptual analysis deliver on this promise? Can it help us understand and explain a *complex* social phenomenon such as law (to use Hart’s own terminology)?

Before I address this question, however, we need to deal with a second form of armchair jurisprudence.

### B. Metaphysical Reduction

Conceptual analysis is not the only endeavour in which legal philosophers have resorted to their own intuitions to formulate metaphysical claims about the nature of law. Some philosophers, such as Andrei Marmor, have explicitly disclaimed conceptual analysis in favour of explanations that aim at what they call ‘grounding reductions’. My goal here, then, is to show that, even if this kind of theory construction is not a form of conceptual analysis, it is still a form of armchair theorising.

Generally speaking, reductive explanations are explanations in which one type of fact or proposition fully accounts for a different type. Thus, to offer a reductive explanation of A in terms of B (ie to reduce A to B) is to say that A can be fully explained or accounted for by B.^19^ Since, in the realm of social ontology, we deal with the *nature* of social facts and institutions, offering reductive explanations in this realm must entail offering *grounding* reductions (not merely semantic ones).^20^ Accordingly, reductive explanations of law must be reductions of law to its *constituent* elements (similar to how the density of a body is rightly reduced to its mass and volume).^21^ This way, general jurisprudence does not and should not revolve around the analysis of concepts but around a purely metaphysical enterprise instead.^22^

So, what are the grounding, constitutive facts of legality? Law’s constitutive facts are ultimately, Marmor argues, facts about ‘how people behave, the kind of beliefs they have about their conduct, and the kind of attitudes and dispositions that tend to accompany those shared beliefs’.^23^ It is *these*, then, that a theory of law should aim to explain. Most importantly, in explaining them, the theorist does not need to rationalise the practice in a way that makes sense to the participants themselves.^24^

Here we have come to the key methodological question for a grounding reduction of law. Even if one accepts both that the aim of legal theory is to determine the grounding facts of legality and that such an aim is not limited by hermeneutic concerns, we still need to explain how precisely we are supposed to go about this. Surely the reason why (at least some) philosophers engage in conceptual analysis is precisely that they think that serious metaphysics needs to proceed by analysing concepts (ultimately because conceptual analysis seems to be, for them, the only legitimate task for philosophy). So, merely observing that the goal of general jurisprudence is to provide metaphysical reductions does not solve the key methodological issue.

Ultimately, if we take Marmor’s intention to dismiss both (i) conceptual analysis and (ii) hermeneutic explanations seriously, I see only two alternatives to interpret what a ‘grounding reduction’ requires methodologically speaking—ie to understand how a reduction of a social phenomenon such as law to its constituent elements *should be done*: the theorist can either work exclusively from his own intuitions (which, in this case, he does not need to assume are those of the participants of the practice themselves) or he can work from our best current empirical scientific findings.^25^

Now, Marmor himself tells us that grounding reductive explanations are concerned with properties or features of *observable* phenomena—properties that can be learned from experience.^26^ Thus, the obvious answer to our methodological question should be that, in performing a grounding reduction of legal phenomena, the theorist must assign our best current scientific findings on the matter a central place. Since grounding relations are learned by observing the world, it is from these findings that theory construction should proceed. So, this is precisely what one would expect to see in Marmor’s legal philosophy.

In fact, however, what we find when we turn to Marmor’s own propositions on the nature of law, even if implicitly, is that theory construction proceeds from the theorist’s *own* intuitions (even though nothing in the strategy of ‘grounding’ would seem to require this). If one were asked to describe what it is that legal theorists do by looking at how Marmor proceeds, one would need to say that the theorist works using his *own* intuitions as the exclusive evidential source for his inferences. In Marmor’s *practice* of legal philosophy, then, we find little suggestion that we should assign our best current science a central evidential role.^27^

Whether the project of analytical general jurisprudence has always been the project of offering grounding reductions of law instead of an analysis of concepts is not something that needs to detain us here. Both projects share the methodological feature relevant for our purposes: the theorist’s *own* intuitive judgments are the *exclusive* evidential source for his inferences. Methodologically speaking, a ‘grounding reductive’ explanation is a distinctive approach to general legal theory only in two senses: (i) the theorist does not need to assume that his own intuitions are those of the participants (since the perspective of the participants themselves might be irrelevant or, in any case, not the ultimate standard against which the correctness of the theory should be tested); and (ii) the theorist does not need to assign *linguistic* intuitions a central place (or any place whatsoever). But precisely because *these* are the only two differences, a ‘grounding reduction’ of law is also a form of ‘armchair jurisprudence’ along with conceptual jurisprudence. Both forms of theory construction proceed *exclusively* from the theorist’s *own* intuitions.

### C. Armchair Jurisprudence and Its Shortcomings

I have been maintaining that the main feature of armchair jurisprudence is the use of the theorist’s *own* intuitions as the exclusive evidential source for his inferences. In the case of conceptual analysis, the theorist typically (though not always) assumes that his own intuitions (linguistic intuitions occupying a central place) are also those of the relevant community.^28^ In the case of grounding reductions, the theorist does not need to assume this, because he does not need to rationalise the practice in a way that makes sense to the participants themselves. In both cases, the theorist constructs general propositions that match or extend in an acceptable way his own intuitive judgements.

Two things must be noted before we move on. First, at least *in its strongest form*, armchair theorising does not aim at synonymy (ie we are not seeking to formulate propositions that are *synonymous* with those that capture our intuitive judgments) but, as Quine put it, merely at ‘approximate fulfillment of likely purposes of the original sentences’.^29^ And second, *in its strongest form*, armchair theorising should rely on some sort of coherentist theory of justification (as opposed to a foundationalist one). Thus, the intuitive judgments with which we start ground our inferences only provisionally—ie though they indeed possess some initial degree of non-inferential justification, they do not have a rock status.^30^ On the contrary, our intuitive judgments might themselves require justification and can be adjusted or abandoned along the way if they conflict with the general propositions that we formulate or with other intuitive judgments.^31^ Thus, at least *in its strongest form*, armchair jurisprudence does not (and need not) assume the validity of some kind of *a priori* knowledge.

We can encapsulate these insights in the following statement: *in its strongest form*, armchair jurisprudence is a type of reflective equilibrium. The theorist, then, should work ‘from both ends’—ie go back and forth between the general propositions that he formulates and his own relevant intuitions—making the adjustments necessary to allow him to arrive at a state of affairs in which both coincide. The justification of the conclusions at which the theorist arrives will be a matter of ‘everything fitting together into one coherent view’.^32^

So what is wrong, if anything, with armchair jurisprudence understood in this, its strongest form? In the following, I want to bring attention to two set of problems that any form of theory construction that relies *exclusively* on the theorist’s *own* intuitions faces.^33^ Ultimately, these problems lead us to the conclusion that anyone who both aims at explaining the nature of a social phenomenon *and* acknowledges that all knowledge must answer to experience must reasonably discharge an obligation to empirically broaden the pool of intuitive judgments on which he relies and find additional evidential sources (beyond intuitions) to support his inferences.^34^

The first set of issues comes from the fact that the theorist works exclusively from his *own* intuitions—intuitions that he assumes are shared by others. This difficulty appears in two different ways. (i) In the case of theories concerned with explaining the hermeneutical character of law, the theorist will have to assume that his own intuitions are those of the participants of the practice. But what reason does one have to assume precisely this? We can expect any one individual to have a host of different intuitions, some of them indeed as participant of a practice, but others as non-participant (critic or observer) or even as participant of a different social practice. How can the theorist ever be sure that the intuitive judgments against which he checks his inferences indeed belong to that *legal* part of the self-consciousness of a *community* (and are not merely his own)?

(ii) The second way in which this difficulty appears also applies to theories that dismiss hermeneutical explanations. Even if our interest is to reduce legality to its grounding elements, so long as we do it from the armchair, we will be working, at best, from the intuitions shared by the members of the particular community of critics or observers to which the theorist belongs, or, at worst, from his own idiosyncratic intuitions. And this poses a problem for both the universalistic project of theories that aim at determining the nature of law for every time and place (ie at identifying the essential properties without which a phenomenon would not be law^35^) and the less ambitious project of theories that acknowledge the contingent character of the nature of law as a *social* phenomenon.

Regarding the former, if we aim at providing explanations that have a universal (or at least general) application, limiting our evidential sources to the theorist’s own intuitions is a questionable move. Even if he is right that his intuitions are shared by other members of the particular social group to which he belongs, there is no guarantee that they will be shared by the members of every group that is *relevant* in providing universal (or sufficiently general) explanations. Such intuitions can, at best, tell us only something about a particular sociocultural context (located in a certain time and place).^36^

Regarding the latter, even if we set aside the universalistic project and embrace more modest ambitions, different groups within one and the same society may have different intuitions about legal phenomena. The problem, then, becomes that of identifying the relevant community, and it appears as the question of whose intuitions count for theory construction in general jurisprudence. Should the theorist work from the intuitions of the community of scientific observers? Or should he work from those of the members of a particular intellectual tradition (eg Marxism, feminism)? Armchair jurisprudence cannot provide an answer to this question using exclusively its own resources.

The second set of issues comes from the lack of legitimacy of using intuitions simpliciter as the sole evidential source when the theorist extends his activity to speculate about the actual character of observable phenomena (something that general jurisprudents are in need of doing if they purport to say something about the nature of a social phenomenon and not just about the meaning of concepts). The problem is that speculating exclusively on the basis of intuitions does not seem to provide us with a legitimate *a posteriori* method for determining the actual character of a social phenomenon. Such a way of proceeding can provide, at best, weak support for any specific conclusions. Hence, excluding other possible evidential sources in formulating our theoretical propositions is evidently a bad strategy. And this problem equally affects immodest forms of conceptual analysis as well as grounding reductions, since they both rely *exclusively* on intuitions to say something about the actual character of the social phenomenon of law.

These two sets of issues show that relying *exclusively* on the theorist’s *own* intuitions is not the best strategy to follow if we aim at providing adequate explanations of the social phenomenon of law. In fact, these problems make one wonder if it would not be a better idea to do away altogether with armchair jurisprudence in attempting to advance our understanding of legal phenomena. This is precisely the conclusion that some legal philosophers have reached, and it has prompted them to argue that we should radically naturalise legal theory. I turn now to this.

## 3. *Radically Naturalised Jurisprudence*^37^

Brian Leiter, one of the most important critics of mainstream legal philosophy in the English-speaking world, has argued that, if we take seriously the goal of advancing our understanding of the social phenomenon of law, then we cannot proceed just from the armchair. Leiter focuses his criticism on conceptual analysis, but insofar as his objections centre around the use of intuitions as the exclusive evidential source in theory construction, they can be extended to any form of armchair jurisprudence (including grounding reductive theories of law).

Leiter tells us that, in order to take adequate notice of the most important implication that Quine’s challenge to the analytic–synthetic distinction had for philosophy—ie that today, analytical truths no longer have a valid domain in the theory of meaning and that all statements must answer to experience^38^—all philosophy ‘must operate as the abstract branch of successful scientific theory’.^39^ This, of course, includes legal philosophy. Thus, if it is to provide us with adequate explanations of *real* legal phenomena, general jurisprudence should not proceed on the basis of ‘intuition-mongering and armchair sociology’.^40^ Instead, it should follow the empirical sciences and resort to conceptual theorisation only when it is useful to facilitate *a posteriori* research about how the world actually works.^41^

In this radically naturalised picture, there are two things that jurisprudence would enable us to do: (i) contribute to the causal explanation of legal phenomena that actually take place in the world; and (ii) elaborate the concept of law and other law-related concepts in a non-arbitrary manner (ie in a manner that does not proceed merely from intuitions and personal observations). A certain concept of law will be vindicated, then, because it is ‘the one that figures in the most fruitful a posteriori research programs, ie the ones that give us the best going account of how the world works’.^42^

Leiter uses the work of Jeffrey A Segal and Harold J Spaeth (ie the ‘attitudinal model’ of adjudication) as an example of these points.^43^ In explaining how Supreme Court adjudication *actually* works, Segal and Spaeth also vindicate, according to Leiter, a hard positivist concept of law according to which pedigree is the exclusive criterion of legality. It is only by presupposing a ‘clear demarcation between the ideological attitudes of judges (which are causally effective in determining their decisions) and the valid sources of law’^44^ that one can describe appellate decision making as underdetermined by law—ie that the attitudinal model can be true and explanatory. Thus, by putting the empirical sciences at the forefront of jurisprudence, we will have (i) contributed to the explanation of *real* legal phenomena and (ii) vindicated a specific concept of law in a non-arbitrary manner. This is the best we can hope for if we are trying to advance our understanding of the actual character of a social phenomenon.

Radically naturalising jurisprudence in this way (ie making it a purely empirical enterprise) seems like the obvious response to the shortcomings of armchair theorising. After all, no one would dare to question today the status of the empirical sciences as the most reliable source of knowledge that we have. Why is it, then, that conceptual analysis (a form of armchair jurisprudence) continues to be the main methodological approach to general legal theory in the English-speaking world?

It is, I think, the existence of an important and well-known distinction that prevents us from embracing a radically naturalised (empiricist) jurisprudence.^45^ This is the distinction between an internal and an external perspective to social practices. There are, of course, many ways of interpreting this distinction,^46^ so we need to do some work to specify the meaning of this claim.

### D. *Normative and Hermeneutic Explanations*

Let us start with the claim put forth by some conceptual jurisprudents that, unlike the empirical sciences, conceptual analysis ‘presupposes a certain story about things—one that is our story—and does not purport to describe the world as it is independently of our stories about things’.^47^ In this picture, conceptual analysis is *supposed* to enable the theorist to account for the internal perspective of participants. This position suggests that if, on the contrary, we followed the method of the empirical sciences *strictly*, we would not be able to account for the fact that the object of our inquiry is *hermeneutical*—one that, as Joseph Raz has put it, ‘plays a role in the way in which ordinary people as well as the legal profession understand their own and other people’s actions’.^48^

A radical naturalist such as Leiter has, of course, an answer to this problem: all we need to do in order to account for the normative and hermeneutic character of a social phenomenon is to reduce the notions of reasons and self-understandings to their psychological, motivational force (ie to the affective dispositions that causally explain our actions and beliefs). Beliefs and actions, then, would be (at least sometimes) *caused* by ‘reasons’.^49^ An empiricist naturalism would thus be able to include *reasons* in its explanations and thereby account for the self-consciousness of participants and the phenomenology of normativity inherent in social practices. Therefore—the reductive naturalist may conclude—that the normative and hermeneutic character of legal phenomena must be accounted for is really no objection at all to a radically naturalistic approach.

The conceptualist may correctly reply here that this is evidently not how the participants of legal practice experience normativity—the practice of giving and asking for reasons. Radical naturalism indeed takes the phenomenology of normativity as a *datum*, but it does not account for such normativity in the way it is experienced by the participants themselves. When participants of normative and hermeneutic practices consider the reasons they have to understand themselves and to act in a certain way, they do not just examine their affective dispositions, measure how much the reasons to which they give rise motivate them and determine whether other reasons would change this level of motivation (ie they do *not* just ask themselves ‘as a matter of fact, what are my affective dispositions here?’).

Instead, participants consider reasons as *justifications* for or against a certain interpretation of their situation, and for or against a certain course of action. It is *this* self-understanding that our conceptual jurisprudent thinks we need to account for. To put it in Scott Shapiro’s terms, legal theory must explain the *intelligibility* of the activity of legal actors. A theory that does not take the internal point of view seriously will render ‘incomprehensible the way legal participants think about their actions’.^50^ Thus, it is not just the fact that individuals have meaningful and normative experiences that is at stake here (as reductive naturalists would have it), but *the way in which* they understand themselves as having them. This is, in fact, what is *distinctively* hermeneutic about hermeneutic practices. And this is precisely what a *reductively* naturalistic epistemology misses.

But the radical naturalist might have one more trick up his sleeve. If we have already accounted for the *fact* that law is a hermeneutic practice and taken the phenomenology of normativity as a *datum*, the naturalist may ask, why in the world should we do so in the terms in which the actors themselves think about their actions?^51^ The naturalist could distinguish, for instance, what actors think they are doing from what actors are really doing, and conclude that it is only the latter that should matter for theory construction. Or, as Leiter has done in relation to moral phenomenology, he could ask why we should take normative experiences at face value instead of explaining them ‘in quite different terms in order to locate this experience within our best picture of how the world works’.^52^ For the radical naturalist, explaining normativity in the vocabulary of causes is indeed all that we need.^53^

At this point, we might think that we just have two competing kinds of explanations (ie an interpretive and a reductively naturalistic one) and no way to rationally choose between them. Radical naturalists, however, do think that we have reasons to choose empiricist explanations. For this kind of naturalist, what ultimately vindicates an empiricist epistemology is the existence of *pragmatic* considerations.^54^ A theoretical proposition about law should be accepted, the strong naturalist tells us, if it figures in or is presupposed by those accounts of how the world works that are the most successful for achieving human purposes. These accounts are those provided by the empirical sciences, whose *a posteriori* research programmes have successfully enabled us to communicate with each other from thousands of miles away, light up our cities at night, send planes into the sky on a regular schedule, and so on. Hence, we should prefer reductively naturalistic explanations because the scientific epistemology on which they rest indeed ‘delivers the goods’.^55^ For a radically naturalised jurisprudence, then, it is our need to predict the future that ultimately vindicates the standards that, in turn, justify any one specific theoretical proposition about law.

Thus we come to the crucial point in this debate between the radical naturalist and someone interested in accounting for the point of view of participants in their own terms. Is there any reason to think that our need to predict the future (a need that is satisfied only through causal explanations) is the only need that we have as human beings? Could it not be the case that our experience of normativity *qua* normativity, and of meaning *qua* meaning, is also providing us with standards that are ultimately vindicated by the fact that they work for us, only that this time in a different, non-predictive manner? Is it not possible that there are interests, other than predicting the future, that give us pragmatic reasons to maintain the irreducibility of normative discourse to empiricist naturalistic explanations? If this is the case—ie if legal and moral talk, for example, does not aim at predicting the future—the way we assess whether such talk works for us cannot be the same as with empirical scientific discourse.

Here, I want to explore two complementary lines of arguments, each with different levels of empirical support, that might suggest that normative and hermeneutic talk is indeed doing important work for creatures *like us*.^56^

The first line of argument is well known in sociological research and goes something like this: the advent of modernity left Western societies in the problematic situation of how to keep them from disintegrating in a world in which comprehensive (theological and metaphysical) views were no longer available for the job.^57^ Normative discourse (ie the practice of asking and giving reasons) and discourse about the self-understanding of a community are occupying this space today. By making the justificatory practices of a community possible, and by providing individuals with a shared cluster of symbols that enables them to mutually comprehend each other when coordinating their aims, normative and hermeneutic talk perform an important integrative function. From a sociological standpoint, then, normative and hermeneutic practices may indeed be working for individuals *like us*.

Three things must be noted regarding this line of thought. First, the argument claims that *only* normative practices can perform this task. The case of market relations is particularly illustrative here. A *non*-normative conception of the market tells us that, as a matter of fact, each agent pursues his own interest in the market with no more consideration for the interest of others than what is necessary to satisfy his own (and expects others to also act in this way).^58^ Sociologists realised very early in the history of the study of capitalism and the division of labour, however, that if the market were *only* this (ie only a *non*-normative sphere of action), it could not make for social relationships of a stable kind—something required for social cohesion.^59^ If this is true, for market relations to perform an integrative function they already must be part of a normative order (as I think they are in any case, if the market is to exclude certain kinds of conduct—an agent cannot do just *anything* to take possession of someone else’s property, for example, even if no negative consequence is expected to follow from this, and someone who steals can hardly be described as *acting in the market*).

Second, the argument does not suggest that *law* is the only mechanism that performs an integrative function—simply because law is not the only normative and hermeneutic practice that we find in modern forms of social organisation. Secular morality (the practice of giving and asking for *moral* reasons), for instance, also performs an integrative function, albeit as a weakly institutionalised cultural pattern and, thus, in a much more fragile manner (and with different aims). As we will see, what matters here is that, to the extent that law *is* such a kind of practice (even if not the exclusive one), an adequate and complete explanation of legal phenomena would need to include reference to normative and hermeneutic properties.

Third, this line of argument does connect the general observation about normative and hermeneutic practices specifically to law—something obviously required to vindicate normative and hermeneutic explanations *in legal theory*. This is done in different ways. Scott Shapiro, for instance, has argued that any normative practice that does not involve a hierarchical structure and the existence of impersonal offices and institutionalised procedures could not, by itself, generate the kind of coordination that is needed for social integration in the face of serious moral disturbances.^60^ Only a legal system can do so^61^—which is not to say that in other, not so seriously problematic, contexts *non*-legal normative practices are not enough to do the job. Axel Honneth, in turn, has argued that the kind of coordination that law enables is that among individuals seeking to retreat from more exacting forms of social interactions.^62^ For Honneth, then, unlike morality, law coordinates precisely that kind of action that is not subject to higher principles or expectations from others.

The second line of argument comes from evolutionary psychology. In reconstructing the natural history of morality, Michael Tomasello has argued that normative practices emerged in the history of evolution in order to enable human beings to engage in complex cooperative enterprises. This development took place in two steps, each prompted by new ecological circumstances and each providing human beings with new skills and motivations.^63^ First, ecological changes forced human beings to forage together to avoid starvation. The success of this collaborative enterprise, however, depended on each partner fulfilling their role, so each was given the ability to call others to task and hold them accountable if they did not act as expected. Such a development laid the grounds, then, for the emergence of *joint* intentionality, with its associated kind of ‘second-personal morality’.^64^

Second, dramatic changes in demographics led early social groups to divide into different sub-groups—groups that remained, however, unified at a more abstract level. The success of collaborative enterprises in these new conditions now required new cognitive skills and motivations that enabled individuals to coordinate their activities with others with whom it was not immediately obvious that they were indeed collaborating. It required, then, putting in place *cultural* conventions, norms and institutions. Thus, this development laid the grounds for the emergence of a *collective* intentionality, with its own associated ‘cultural and group-minded, objective morality’.^65^

Note that there are many possible ways of combining the sociological and the evolutionary stories. One could imagine, for example (as has often been suggested), that ever more dramatic demographic changes accompanied by accelerating processes of globalisation have transformed cultures dominated by tradition, religious values and comprehensive views of the world into secular ones centred around the individual. Thus, the kinds of normative practices capable of enabling coordination, cooperation and, ultimately, social integration have also evolved accordingly. Because religious practices and practices of *traditional* morality are no longer able to perform these tasks (simply because they are no longer sufficiently shared among the members of pluralistic communities), today we require normative systems formulated at a sufficient level of abstraction. And this is precisely what a modern legal system is supposed to provide.

For our current purposes, however, what matters is that both stories suggest that normative and hermeneutic talk is working for us. These modes of discourse are the product of developmental processes in which human beings have acquired specific skills and motivations exercised in particular kinds of social practices: normative and hermeneutic practices. Such practices provide their participants with a distinct vocabulary (the vocabulary of reasons) that enables them to coordinate their actions and, ultimately, to be part of complex cooperative enterprises.

If this is the case, in understanding normative and hermeneutic phenomena (such as law), we cannot dispense with explanations that include reference to normative and hermeneutic properties. These kinds of explanations are useful for human purposes because they contribute to our understanding of this (not previously available) vocabulary—ie how to use it, the entitlements and responsibilities that we have in light of previous commitments made in such a vocabulary (by elaborating relations of consequence and incompatibility), and so on. This way, they also help us improve its use and train others to use it.

Thus, if, as a normative and hermeneutic practice, law is doing integrative work by enabling the members of a community to coordinate their action and engage in complex cooperative enterprises, then legal theory cannot just dismiss what is *distinctively* normative and hermeneutic about it—ie it cannot reject explaining law in the terms in which the actors themselves think about their actions. The very function that the social practice of law performs depends on the successful exercising of the skills that it makes available for those who participate in it, and this is precisely what explanations that refer to normative and hermeneutic properties contribute to. Normative and hermeneutic explanations would thus be vindicated.

That explanations that include reference to normative and hermeneutic properties are useful for human purposes should come as no surprise. If one accepts the pragmatic argument that the kind of explanations that the empirical sciences provide is ultimately vindicated by our need to predict the future, one must also accept explanations of these sciences’ normative standards couched in terms that are useful for those who use them as *standards* (those kinds of explanations, for instance, that advisors give their advisees about how research must be done in a certain field, or that textbooks on the methods of some specific empirical science contain^66^). *These* explanations will have to refer to normative properties. Hence, even within empirical scientific practice, there is value to an explanation that takes the point of view of the participants seriously—ie that accounts for the self-understanding of those engaged in the scientific enterprise themselves. When asked why he takes a hypothesis as proven, the scientist does not just answer with a causal explanation of his belief; he answers with what he considers to be reasons that *justify* this belief in front of the scientific community.

Because the practitioner must understand epistemic norms as *norms* (his own activity as norm-governed activity), explanations formulated in the language of norms are useful for him: they show him how to use the epistemic standards of his discipline *properly*, how to properly make inferences from appropriate premises, and so on.^67^ In fact, explanations as to how to use the respective *normative* vocabulary *help* the empirical sciences to function as our best predictive tool in the first place.^68^

The reductive naturalist could reply here, ‘but we have always conceded that causal or nomic explanations contain their own normative standards; what we claim is that those standards are vindicated by their success in satisfying our interest in predicting the future’. But my point here is precisely that, once you have vindicated the normative (epistemic) standards of explanations formulated exclusively by reference to nomic or descriptive properties, you have also already vindicated normative explanations that tell participants of such a practice what follows from what and what is incompatible with what (what moves are indeed *legitimate* in this language game), and so too what one’s entitlements and responsibilities are, given one’s previous commitments. Whatever it is that requires the former also requires the latter. In much the same way, then, whatever it is that requires the existence of normative and hermeneutic practices also vindicates explanations that refer to normative and hermeneutic properties.

### E. Interests and Attitudes

The argument of the previous subsection rests on an empirical question—the question of whether normative and hermeneutic practices are indeed working for us by enabling social integration and, thus, complex cooperative enterprises. Though there currently is some empirical support for answering this question affirmatively (Tomasello, for instance, offers phylogenetic and ontogenetic evidence from experiments with great apes and experiments related to the socialisation processes of children to support his evolutionary story), the task of providing sufficient evidence for this claim is a monumental one, and something I could not hope to do within the scope of this article. There is, however, one point that I need to touch upon before I move on.

In dealing with the legitimacy of a strictly empirical scientific epistemology, Leiter seems to suggest that the burden of proof is much higher for pragmatist arguments that would vindicate explanations couched in a normative and hermeneutic language than for pragmatist arguments that vindicate empirical scientific explanations:

The reasons for being a naturalist in the first place are … pragmatic ones, that almost everyone—including the antinaturalists—actually accept in practice. Naturalism thus makes its claim on us in virtue of its resonance with our attitudes, our practical interests in coping with the future course of our experience in the world.^69^

And again:

Someone might, of course, prefer more moral, spirit, and gustatory facts, and the like, in their ontology, but that is not, by itself, an argument against naturalism, unless one thinks the epistemic norms that license belief in such facts answer to equally or more important practical attitudes of creatures like us. The naturalist, to be sure, noting the extent to which all of us are invested in naturalistic norms because they work so well in coping with the future course of experience, might then point out the pressures created by consistency—though that, too, is an epistemic attitude that is also not epistemically obligatory.^70^

It is suggested that there is no great need to offer evidence to support the pragmatic argument that vindicates empirical scientific explanations because this kind of explanation resonates very deeply with our widely shared attitudes. It is so obvious that we all share the attitudes that vindicate empiricist standards that it would be almost superfluous to require evidence of their existence—in a way that it would not be when it comes to the attitudes that would vindicate normative or hermeneutic explanations.

I do not wish to question here the existence of such widely shared attitudes that resonate with empiricist naturalistic explanations (doing so seems absurd today). However, if what licenses us to *assume* the existence of these attitudes is the extent to which we indeed engage in predictive practices, there are similar reasons to assume the existence of attitudes that resonate with normative and hermeneutic explanations: we do indeed engage in practices of giving and asking for reasons for belief and action, and if not to the same extent, to a very similar one.

In order to be clear about what I mean here, we need to distinguish two possible attitudinal objects: (i) the interest in what a practice is doing for us; and (ii) the normative standards with which the practice works. Leiter himself is ambiguous on this point, and though the strength of his argument rests on the former being the object of our attitudes, sometimes he suggests that it is, in fact, the latter.^71^ Once we make this distinction, we can see that it is possible for us to widely share an interest in making complex cooperative enterprises and social cohesion possible, together with the practical attitudes towards that interest, and disagree on the specific standards that should guide decision making within the practice supposed to satisfy such interest.

Thus, when I say that there are reasons to assume the existence of attitudes that resonate with normative and hermeneutic explanations given by the fact that we do engage in normative and hermeneutic practices, I am referring to a kind of second-order convergence. Though it is true that, here, there is much less agreement on the content of *particular* propositions and on the *specific* norms and principles that should serve as standards for justification, it is also true that, at least in modern, pluralistic societies, individuals converge on resolving disagreements through the general practice of giving and asking for reasons (particularly *legal* reasons), as opposed to engaging in naked expressions of power. We can certainly give causal, psychological explanations for this convergence, but what matters for our purposes here is that, if this is true, advancing our understanding of the vocabulary of this practice is vindicated precisely by considerations of the pragmatic kind that Leiter suggests apply to scientific discourse: interests and the attitudes that accompany them.

Though the two stories that I have offered here (ie the sociological and the evolutionary stories) are themselves couched in observational language, they also suggest how normative and hermeneutic talk might be working for us. Thus, it would be wrong to conclude from the former fact that causal or nomic explanations are all we need to account for normative and hermeneutic phenomena. That these practices have provided us with new skills to cope with our natural and social environment is all we need in a pragmatic story, first, to ‘bootstrap ourselves out of nature and into normativity’,^72^ and second, to vindicate explanations couched in the language that those practices themselves enable their participants to use.

By the same coin, however, nothing prevents us from accepting a *weak* form of naturalism. In fact, the pragmatic considerations that vindicate normative and hermeneutic talk are perfectly compatible with the claim that normative and hermeneutic practices have emerged *from* nature (evolutionary explanations even require it). In this kind of naturalism, the cognitive and practical capacities of all sentient organisms are placed on a continuum: from the hunting skills of the animal predator to the discursive activity of contemporary human beings and the sophisticated theorisation about the nature of the world in which scientists and philosophers engage, they are all conceived as the product of adaptative mechanisms to a continuously evolving environment.^73^ Hence, though they indeed grant language users qualitatively distinct abilities that open new possibilities not previously available to them,^74^ ultimately, *all* discursive practice also emerge from nature.

Most importantly, since, in this framework, the use of any particular language is ultimately vindicated by the contribution that it makes to the satisfaction of some human need or interest—a contribution that will, of course, depend on the resistance of the objective world^75^—one would expect that different languages have developed from different processes of adaptation and, thus, perform different functions that respond to different needs. This way, such naturalism is what we could call, following Huw Price, a form of *subject* naturalism that does not necessarily entail *object* naturalism—the view that no philosophical claim can conflict with the fact that *we*, humans and speakers, are natural creatures, but for which not every form of speech is representational. And precisely because a plurality of kinds of speech would reflect *just* the different roles that different language games perform in our lives (some of them non-representational) given different processes of adaptation, such a form of naturalism would *not* require us to subscribe to the existence of any ‘promiscuous ontology’ whatsoever.^76^

## 4. The Methodological Challenge

If, in understanding and explaining law, we cannot dispense with what is *distinctively* normative and hermeneutic about the practice, does this mean that we must go back to conceptual analysis? We have already pointed out the problems that a jurisprudence that works exclusively from the theorist’s own intuitions has for giving adequate explanations of the social phenomenon of law. Even if the way we take the self-understanding of the practice into account is by determining the meaning of *our* concept of law, the theorist cannot proceed *exclusively* from his *own* intuitions. If this concept is really *ours* (ie it is one shared by a particular linguistic community), the theorist must engage in some sort of empirical investigation of intuitions. This is precisely what experimental jurisprudence has attempted to do.^77^

But even though we welcome experimental jurisprudence, it can hardly be the whole story.^78^ There are two reasons for this. The first is that the problem of arbitrariness appears for the theorist not only in determining the specific intuitions applicable in possible cases, but in formulating these possible cases in the first place (ie in designing thought experiments). Though experimental jurisprudence has offered a solution to the former, it seems to have been silent about the latter. If the theorist is to avoid arbitrariness, however, he should keep his own discretion in selecting (an initially unlimited number of) possible cases in check.

Now, experimental jurisprudents could easily correct for this by letting the design of thought experiments be guided by what our best current scientific findings on the matter tell us that we should prioritise. But there is a second, more important, reason as to why experimental jurisprudence cannot be the whole story: it is that, although, as a practice, it of course contains a self-understanding, the social phenomenon of law cannot be reduced to it. Even though law has no existence independently from human beings’ activity, there are aspects of this activity that are neither conscious nor implicit in the participants’ self-understanding of their actions. General jurisprudents, then, must understand the social phenomenon of law as also having a ‘life’ that can be studied from the perspective of a non-participant. And this is what an empiricist naturalism actually gets right. In this sense, general legal theory must account for two kinds of viewpoints external to the practice: (i) the perspective of the social scientist who studies law causally; and (ii) the perspective of participants of other social practices who examine law *critically*. While the participant *qua* participant takes only (and is, in fact, defined by) an internal perspective, the theorist must navigate between both an internal and an external one.^79^

Therefore, in advancing our understanding of law as a *complex* social phenomenon, the challenge for legal theory is to avoid falling into two pitfalls. On the one hand, in accounting for either the self-consciousness of participants or the constitutive elements of law, we need to avoid working exclusively from the theorist’s own intuitions. On the other hand, though we must indeed account for the causal life of the practice, we need to avoid reducing legal theory to the role of mere facilitator of empirical research. Neither armchair jurisprudence nor radical (empiricist) naturalism, then, looks entirely promising. Is there a way out of this alternative? Or, to put it differently, is there a way to correct their shortcomings without giving up what each position indeed gets right?

What we need is, I claim, a methodological approach that can *legitimately* move between the external point of view characteristic of the social scientist and the critic and the internal point of view of participants—all the while maintaining the unity of its object of study.^80^ If, methodologically, we are to do justice to this complexity, the theorist must work, so to speak, from four rather than two ends:^81^ the general propositions that he formulates; the judgments that participants of the practice intuitively make; the best empirical observations of the social phenomenon at stake (paradigmatically provided by the social sciences); and the perspective of other social practices external to law. Though the theorist must take both the perspective of the participant and that of the external observer (either scientist or critic) into account, his own perspective can be reduced to neither. Only this way can he hope to select ‘adequate’ possible cases, include all the relevant *data* available and extend the pool of evidential sources to the full extent of our possibilities.

At this point, someone could object that there is no reason why we should want one and the same discipline to adopt a methodological dualism like the one I am proposing here.^82^ It might be true that law cannot be reduced to either the self-consciousness of participants or the object of empirical scientific inquiry and still be appropriate, our objector could say, to appeal to an academic division of labour. Mainstream jurisprudents, for example, would be particularly prone to argue that a metaphysical investigation of the nature of law is all there is to general jurisprudence—whereas the study of the contingent properties of particular legal systems should be left to the social sciences.

To this objection, my reply is that embracing dualism as a methodological maxim enables us to attain a better understanding of the social phenomenon with which we are concerned—ie it enables us to give better, more complete and thus more adequate explanations of it. The answer comes in two steps. First, we note that a *general* theory of law is not merely concerned with metaphysics, in the same way in which, when biologists produce theories of evolution, explaining the constitutive elements without which a certain phenomenon would not be evolution is *not* their main concern. They are, instead, attempting to say something about the actual character of a certain part of the world, and this includes all its relevant contingent properties.

Second, once we acknowledge that our concern is with the phenomenon in its entirety—with some of its properties being graspable only from the self-understanding of participants and others only from the perspective of external observers—the question of whether we should prefer partial explanations (that rely on an academic division of labour) or complete ones hinges on epistemic values such as consilience, unification and systematisation.^83^ Thus, a methodological approach that provides explanations that unify different aspects of the same phenomenon should be preferred to any methodological approach that provides us only with half of the story. And the kind of dualism that I propose here would provide us with explanations that are indeed more consilient than those provided by any *methodological* monism because they capture and unify facts ‘taken from more than one domain’,^84^ and they do so without reducing one class to the other.^85^

Thus, if general jurisprudence is seriously concerned with advancing our understanding of the *complex* social phenomenon of law in its entirety, it must be able to explain both how the kind of abilities that law makes available to us are to be used *and* how actual patterns of exercise of such abilities *causally* interact with the world and with other social practices, institutions and structures.

## 5. Conclusion

With the methodological challenge in view, the following dilemma arises. If one accepts that to advance our understanding of the social phenomenon of law general jurisprudence must account for its complexity, then it seems that, for a long time now, mainstream general jurisprudents in the English-speaking world have been missing an important part of the puzzle. As a form of armchair jurisprudence, conceptual analysis, the main methodological approach to general legal theory, is simply incapable of giving us an adequate account of this complexity. What conceptual analysis may give us is just a small part of the picture: it might help us understand general legal thought and talk (though even to accomplish this goal it needs to be supplemented with an empirical investigation of intuitions). We are, then, faced with an alternative: we either redefine the object of general jurisprudence by looking at what self-identified general jurisprudents have been really doing in the last decades (ie we explain what general jurisprudence is by reference to *that*) or accept that the discipline has been led stray from its legitimate task (a goal explicitly embraced by HLA Hart) and move to determine what is necessary to get back on track (which, I have argued, cannot consist in just *radically* naturalising jurisprudence).

While philosophers like David Plunkett and Scott Shapiro have taken the former path and thus relegated *general* jurisprudence to a branch of metanormative inquiry,^86^ here I am proposing that we should take the latter. In other words, while the label of metanormative inquiry might seem right for *conceptual* jurisprudence, *general* jurisprudence, I claim, does not need to be conceptual.

## Footnotes

1 Thus, I use the label ‘armchair’ in the same way in which discussions about general philosophical methodology use it.

2 Thus, theory construction will be intuition-deploying without being an armchair endeavour so long as the theorist engages in some sort of empirical investigation of shared intuitions (or, alternatively, so long as intuitions are not his sole evidentiary source).

3 Some theorists proceed from the armchair even though they claim to have taken notice of the case that Quine made for the proposition that all forms of knowledge must ultimately answer to experience. Whether this is indeed a tenable position depends on what we understand that we do from the armchair. For some arguments in favour of the compatibility of naturalism with (some forms of) armchair philosophy, see Hilary Kornblith, ‘Philosophical Naturalism’ in Herman Cappelen, Tamar Szabo Gendler and John Hawthorne (eds), *The Oxford Handbook of Philosophical Methodology* (OUP 2016) 154.

4 Or, as Himma puts it, by determining ‘the properties that constitute something as falling within the reference of a concept-term’. Kenneth Einar Himma, ‘Conceptual Jurisprudence. An Introduction to Conceptual Analysis and Methodology in Legal Theory’ (2015) 26 Revus 70.

5 As WV Quine famously put it, ‘Meaning is what essence becomes when it is divorced from the object of reference and wedded to the word’. WV Quine, ‘Main Trends in Recent Philosophy: Two Dogmas of Empiricism’ (1951) 60 The Philosophical Review 22. After the discredit of logical positivism and the Wittgensteinian turn to practices, these properties (which constitute something as falling within the reference of a concept) came to be understood as depending on linguistic *usage* and not simply on semantic relationships.

6
*Strict* linguistic analysis allows us to determine the meaning of words or expressions and the criteria for their application by identifying shared intuitions about their use. Though this might serve fine as a starting point to investigate the nature of law, in the view of most conceptual jurisprudents, conceptual analysis involves a much more substantial enterprise than just tracking and reporting patterns of word usage.

7 He did so both in the initial passages of *The Concept of Law* and in the Postscript. See HLA Hart, *The Concept of Law* (2nd edn, OUP 1994) 17, 239.

8 Almost half a century after *The Concept of Law* first appeared, Joseph Raz echoed Hart’s remarks in insisting that the object of explanation of legal theory was ‘the nature of law’ as a social institution. See Joseph Raz, *Between Authority and Interpretation: On the Theory of Law and Practical Reason* (OUP 2009) 17, 20.

9 ibid 24.

10 As is commonplace now, here I am following the terminology put forth by Frank Jackson. See Frank Jackson, *From Metaphysics to Ethics: A Defence of Conceptual Analysis* (Clarendon Press 1998) 42.

11 The thesis that this mode of conceptual analysis is different from mere linguistic analysis is not entirely uncontroversial—it depends on the possibility of distinguishing between concepts and the terms that we use to designate them. One way in which philosophers have formulated this distinction is in terms of surface and deeper meaning. While lexicographic research would allow us to determine surface meaning, concepts also involve a deeper meaning that goes beyond the core, explicit features of our linguistic practices. Determining this deeper meaning, then, may require the use of intuitions about features of the concept of which ordinary language users are not cognisant. Thus, as Himma puts it, ‘when we analyze a concept, we break it down into simpler constituents so as to display its logical structure and give an explanation of its content that transcends, but incorporates, its ordinary lexical meaning’. Himma (n 4) 74. Similarly, Stephen Perry has pointed out that ‘Typically, the philosophical analysis of a concept attempts to make explicit what the theorist claims is in some sense already implicit in our common understanding. This can take the form of drawing attention to propositions that the theorist argues are either implicitly presupposed or necessarily entailed by the concept’s having the content that it has.’ Stephen Perry, ‘Hart’s Methodological Positivism’ (1998) 4 Legal Theory 448. Against the possibility of distinguishing concepts and the words that we use to designate them, see Andrei Marmor, ‘Farewell to Conceptual Analysis (in Jurisprudence)’ in Wil Waluchow and Stefan Sciaraffa (eds), *Philosophical Foundations of the Nature of Law* (OUP 2013) 212.

12 Himma (n 4) 70.

13 Jackson (n 10) 43.

14 See Hart’s Preface to his *The Concept of Law*. Hart (n 7) v.

15 Langlinais and Leiter offer an interpretation of Hart’s method precisely in this sense, based on the fact that law is constituted by linguistic practices. See Alex Langlinais and Brian Leiter, ‘The Methodology of Legal Philosophy’ in Cappelen, Gendler and Hawthorne (n 3) 676.

16 ibid.

17 Aleardo Zanghellini, ‘A Conceptual Analysis of Conceptual Analysis in Analytic Jurisprudence’ (2017) 30 CJLJ 485.

18 The fact that there is not one but multiple conceptions of what conceptual analysis entails has been approached from different perspectives. Natalie Stoljar, for example, argues that there have been in fact three different projects under the umbrella of ‘conceptual analysis:’ (i) a conceptual project, delivering what she calls a ‘manifest concept’; (ii) a descriptive project, delivering an ‘operative concept’; and (iii) an ameliorative project, delivering a ‘target concept’. See Natalie Stoljar, ‘What Do We Want Law to Be? Philosophical Analysis and the Concept of Law’ in Waluchow and Sciaraffa (n 11) 232. I thank one of my anonymous referees for bringing this to my attention.

19 Andrei Marmor, *Foundations of Institutional Reality* (OUP 2023) 33.

20 ibid 38.

21 General jurisprudence is, then, an enterprise whose goal is and should be ‘to show that a distinct type of phenomenon is actually constituted by, and fully reducible to, some other, more foundational type of phenomenon’. Marmor, ‘Farewell’ (n 11) 215.

22
*Pace* Marmor, it is not entirely clear to me that reducing something to its constitutive elements in this way does not entail a form of conceptual analysis. Indeed, Marmor himself points to Hart’s as an example of a reductive theory of law, and to the extent that Hart’s aim was to uncover grounding relations between people’s conduct, beliefs and attitudes and certain social facts, and between those social facts and law, he did rely on conceptual analysis to support his inferences—ie he relied on the ‘widespread common knowledge that any educated man has’ of the way we think and talk about law and law-related phenomena. See Langlinais and Leiter (n 15) 676. Moreover, if conceptual analysis is different from mere linguistic analysis and includes experientially informed speculation about the properties of the things of which the concepts are concepts as well as direct empirical observation (as some legal philosophers understand it to include), then the activity of formulating the constitutive, more foundational elements of law could obviously be a form of conceptual analysis. Ultimately, however, this is not something that need detain us here.

23 Marmor, ‘Farewell’ (n 11) 214.

24 Marmor, *Foundations* (n 19) 126. Thus, according to Marmor, not only is a metaphysical explanation *not* conceptual, it is also *not* hermeneutical.

25 There is nothing strange in suggesting that metaphysicians should keep an eye on the best current science on the topic, and even use scientific results as a ‘positive guide’. See Daniel Nolan, ‘Method in Analytic Metaphysics’ in Cappelen, Gendler and Hawthorne (n 3) 164.

26 Thus, they are not concerned with the *a priori* necessary conditions for something to be law. See Marmor, ‘Farewell’ (n 11) 216, 223.

27 Perhaps Marmor would indeed be willing to assign the results of empirical science a central place.

28 As we will see, this is a shortcoming that experimental jurisprudence seeks precisely to correct.

29 WV Quine, *Word and Object* (Martino Publishing 2013) 224.

30 For the difference between ‘rock’ and ‘ground’ status of intuitions, see Jonathan M Weinberg, ‘Intuitions’ in Cappelen, Gendler and Hawthorne (n 3) 290.

31 See Yuri Cath, ‘Reflective Equilibrium’ in Cappelen, Gendler and Hawthorne (n 3) 228.

32 John Rawls, *A Theory of Justice* (revised edn, Belknap Press 1999) 19.

33 I take it that these problems are well known. Thus, in this section, I am building upon Brian Leiter’s convincing objections to the role of intuitions and conceptual analysis in legal philosophy. See Brian Leiter, *Naturalizing Jurisprudence: Essays on American Legal Realism and Naturalism in Legal Philosophy* (OUP 2007) 183–99.

34 And distinguishing essential from merely contingent properties of social phenomena will not deliver us from this fate. In fact, if one subscribes to the view that legal theory is not supposed to provide us with any kind of *a priori* necessary knowledge (most likely because such knowledge is impossible), one cannot but wonder what work this distinction is supposed to be doing at all. The knowledge of essential properties answers to experience as much as the knowledge of contingent ones. This is why it is so strange that Marmor concedes both (i) that ‘essential properties of social practices are properties or features of observable and learnable activities, not of language or concepts’ and (ii) that it is ‘mistake to equate essential properties of X with a priori necessary conditions’, and still thinks that whether a property is essential depends on a counterfactual which can be determined by just thinking about and imagining possible worlds (ie that ‘[a] feature of a social practice is essential to it if without it the practice would either not have existed at all or would have been radically different from what it is’). Marmor, ‘Farewell’ (n 11) 223–5. What exactly is it that thinking and imagining from the armchair enable us to legitimately attain knowledge of, if not of *a priori* necessary conditions or properties of language or concepts?

35 Which is the explicit project of philosophers such as Joseph Raz and Scott Shapiro. See Raz, *Between Authority and Interpretation* (n 8) 25; Scott Shapiro, *Legality* (Harvard UP 2011) 9. It should be said that Hart was much more cautious here, and that he should probably not be read as a hard essentialist (or universalist, for that matter). Hart himself noted in the Postscript that his concern was with the salient features of a modern municipal legal system. See Hart (n 7) 240.

36 Raz, in fact, does not have any problem with this conclusion: ‘in legal theory there is a tension between the parochial and the universal. It is both parochial and universal. On the one hand it is parochial, for it aims to explain an institution designated by a concept that is a local concept, a product of modern Western civilization. On the other hand it is universal theory for it applies to law whenever and wherever it can conceivably be, and its existence does not presuppose the existence of its concept; indeed it does not presuppose the existence of any legal concept.’ Raz, *Between Authority and Interpretation* (n 8) 38. But the question remains: assuming that Raz’s parochial concept of law really reflects at least someone’s (ie some group’s) intuitions, do they really reflect all the intuitions that are relevant for elaborating *the* concept of law of ‘modern Western civilization’? Or could they be just the intuitions of a certain place or social group?

37 In what follows, I use the expressions ‘radical naturalism’, ‘strong naturalism’ and ‘empiricist naturalism’ interchangeably to refer to a kind of reductive naturalism according to which all valid forms of knowledge must be formulated in strictly observational language (ie by reference to nomic or descriptive properties).

38 Leiter, *Naturalizing Jurisprudence* (n 33) 175.

39 ibid 180.

40 ibid 133.

41 Similarly, Dan Priel has suggested that, even in the best scenario, philosophical investigation should be concerned only with the general question about the nature of social constructions. Anything beyond that—such as the question of the nature of law—would involve ‘the mistaken attempt of filling in the empirical (“sociological”) details about a specific social construction by way of philosophical reflection’. Dan Priel, ‘Law as a Social Construction and Conceptual Legal Theory’ (2019) 38 Law and Philosophy 276. Thus, anyone who thinks that law is a social construction ‘should rephrase claims currently presented as conceptual truths about the nature of law as empirical hypotheses about legal practice in a certain time and place, or as prevailing attitudes about law. If empirically validated, these findings may well be important and illuminating; and they may be called, if one so wishes, a “theory of law”. But the word “theory” will be used here in a sense much closer to the way it is used in most scientific contexts, namely as a way of organizing a body of empirical data, not something that precedes such data.’ ibid 278.

42 Leiter, *Naturalizing Jurisprudence* (n 33) 134.

43 In short, Segal and Spaeth explain US Supreme Court decisions as the product of the ideological attitudes of judges. See Jeffrey A Segal and Harold J Spaeth, *The Supreme Court and the Attitudinal Model* (CUP 1993); Jeffrey A Segal and Harold J Spaeth, *The Supreme Court and the Attitudinal Model Revisited* (CUP 2002).

44 Leiter, *Naturalizing Jurisprudence* (n 33) 188.

45 Here, then, I am in direct disagreement with Langlinais and Leiter, who think that the strongest objection to a naturalised jurisprudence comes from the metaphysical account of law as a social construct and not from the need to account for the self-understanding of participants. See Langlinais and Leiter (n 15) 679.

46 HLA Hart himself offered us one that, according to Scott Shapiro, does not equate the internal point of view with the point of view of participants (since participants can also adopt the point of view of Holmes’s bad man). See Scott Shapiro, ‘What Is the Internal Point of View?’ (2006) 75 Fordham L Rev 1159.

47 Himma (n 4) 74.

48 Joseph Raz, *Law and Morality* (Routledge 2017) 321. Unlike other objects of inquiry, ‘The identification of a certain social institution as law is not introduced by sociologists, political scientists, or some other academics as part of their study of society. It is part of the self-consciousness of our society to see certain institutions as legal. And that consciousness is part of what we study when we inquire into the nature of law.’ Raz, *Between Authority and Interpretation* (n 8) 31.

49 Though the relation between norms, self-understandings and affective dispositions is a complex one, here I will take the hermeneutic and normative characters of a phenomenon as one and the same feature. Though I cannot justify this here, the reason is that, because the practice of giving and asking for reasons for action (in this case, reasons that have their source in law) is part of the self-understanding of participants, the normative character of law follows from its hermeneutic character— participants understand themselves as beings that give, ask, consider, etc reasons for action. And because, in deploying concepts (legal concepts in this case) to make sense of their world and their actions, individuals are already involved in a normative practice (what they are entitled to conclude depends on *shared* meaning), hermeneutic practices are always normative ones. My point, then, is that the structure of this experience, ie the phenomenology of normativity, in short, cannot be grasped from a radically naturalistic perspective.

50 Shapiro, ‘What Is the Internal Point of View?’ (n 46) 1167.

51 Similarly, though without taking this insight to its empiricist consequences, Marmor thinks that ‘We must explain the rationalizing functions of the practice, what makes participants engage in it, but not necessarily in terms that the participants would come to recognize as their own, as their own reasons to engage in the practice or value various aspects of it’. Marmor, *Foundations* (n 19) 126.

52 Leiter, *Naturalizing Jurisprudence* (n 33) 237.

53 This is why this radical or strong naturalism is a *reductive* naturalism. According to it, we only admit to our ontology that which the best empirical scientific findings require us to admit. Leiter is explicit on this. All that really exists is that which can be explained in the vocabulary of causes, and the only real knowledge is that which comes from the method of the natural sciences. Brian Leiter, ‘Normativity For Naturalists’ (2015) 25 Philosophical Issues 69.

54 Leiter refers to two pragmatist arguments. According to the first one (one put forth by the classical legal realists but that is not clear Leiter himself endorses), a theoretical proposition about law (more specifically, about adjudication) should be accepted if it is useful to lawyers. In advising their clients, lawyers are interested in predicting how courts will decide. Thus, explanations that more accurately predict the behaviour of judges should be preferred to those that have very little predictive power. According to the second pragmatic argument that Leiter points out (one which, unlike the first one, he does explicitly endorse), a theoretical proposition about law should be accepted if it figures in or is presupposed by those accounts of how the world works that are the most successful for achieving human purposes. I do not make much of the former here since I take it that the real argument is the latter.

55 Leiter, *Naturalizing Jurisprudence* (n 33) 149.

56 I am quick to admit that these two lines of argument need to be developed much further than I can do here, but I hope that my treatment of them will at least provide us with the raw materials to see why adequate, complete explanations of the social phenomenon of law cannot just eschew reference to normative and hermeneutic properties.

57 A problem all too familiar already to Émile Durkheim and Max Weber.

58 Though it is not my attention to discuss ‘Das Adam Smith Problem’ at any length here, this is precisely how Smith’s description of commercial exchange in the *Wealth of Nations* (and elsewhere) has been *typically* interpreted: ‘It is not from the benevolence of the butcher, the brewer, or the baker that we expect our dinner, but from their regard to their own interest. We address ourselves, not to their humanity, but to their self-love, and never talk to them of our own necessities, but of their advantages.’ Adam Smith, *An Inquiry into the Nature and Causes of the Wealth of Nations*, I.2.

59 Already in *De la division du travail social*, Durkheim himself argued that, since the continuity of harmonious contractual relations does not occur spontaneously, it in fact requires the existence of law to ensure that the conditions of cooperation remain fixed. If this were not the case, contractual relationships would be plagued by constant quarrels and conflicts, and could not, thus, constitute the basis for social cohesion. This is how Durkheim himself puts it: ‘For where interest alone reigns, as nothing arises to check the egoisms confronting one another, each self finds itself in relation to the other on a war footing, and any truce in this perpetual antagonism cannot be of long duration. Self-interest is, in fact, the least constant thing in the world. Today it is useful for me to unite with you; tomorrow the same reason will make me your enemy. Thus such a cause can give rise only to transitory links and associations of a fleeting kind.’ Emile Durkheim, *The Division of Labor in Society* (Free Press 2014) 160–1.

60 Shapiro, *Legality* (n 35) 165–9.

61 ‘Legal institutions are supposed to enable communities to overcome the complexity, contentiousness, and arbitrariness of communal life by resolving those social problems that cannot be solved, or solved as well, by nonlegal means alone.’ ibid 171.

62 The fact that rights and legal norms work as conversation stoppers or, to put it more technically, are supposed to prevent considerations of an extralegal nature from entering the process of deliberation expresses the idea that law provides individuals with a space to which they can retire from more exacting forms of communication. Axel Honneth, *Freedom’s Right: The Social Foundations of Democratic Life* (Columbia UP 2013) 73.

63 Michael Tomasello, *A Natural History of Human Morality* (Harvard UP 2018) 3.

64 ibid 4.

65 ibid 5. Tomasello has supported this story with phylogenetic and ontogenetic evidence from experiments with great apes and experiments related to the socialisation processes of children.

66 Neither the advisor nor the textbook tells students merely that this is how we have (causally) come to believe something, or merely that this is likely how scientists in the field will proceed in the future.

67 If we explained scientific practice exclusively by reference to nomic or descriptive properties, we would be, at best, formulating predictions about what scientists will do. But this is clearly not the only knowledge that scientists themselves, as participants of the scientific enterprise, find useful (not even in those cases in which their topic of study is something like the sociology of scientific knowledge).

68 The fact itself that ‘the deliverances of naturalistic norms generally work well for creatures like us’ depends on participants of the scientific practice conceiving of their activity as governed by norms and as users of a normative vocabulary. See Leiter, ‘Normativity For Naturalists’ (n 53) 72.

69 ibid 68.

70 ibid 73.

71 He specifically suggests this in the following passage: ‘We can easily imagine a world—since such worlds have existed—in which perceptual evidence is not treated as even defeasibly veridical, in which the so-called “scientific method” is dismissed, and in which the dominant epistemic values are what the holy book says or what the holy leader declares. Worlds governed by such epistemic norms tend to have features we modern, postEnlightenment folk find unpleasant, but that is, itself, another attitudinal response. If enough of our fellows share our attitudes, then darkness recedes, and Enlightenment triumphs. But those are facts about people’s attitudes, as influenced by their pleasures and pains, their inclinations and aversions, their loves and hatreds, and not about real normativity.’ ibid 76. Note that the reason given here as to why modern empirical science is to be preferred over medieval practices is not that the former is better at formulating predictions, but that we have a certain attitude towards them. In this version of the argument, if enough of our fellows accept the norms of a practice, then the use of its standards will be vindicated. And because our own historical circumstance is one in which, the argument goes, we widely share an attitude of acceptance towards natural scientific practices but we do not share it towards normative and hermeneutic ones, it would follow that the latter cannot be vindicated in this manner. Thus, in this version of the argument, the object of the relevant attitudes has changed. It turns out that what vindicates natural scientific practices is not that we all widely share a practical interest in coping with the future and accept this interest as a reason for vindicating natural scientific explanations (ie we share an attitude of acceptance towards *this* interest); it is an attitude towards the standards themselves that are supposed to satisfy that interest. At this point, however, one would have to wonder if Leiter’s ‘reasons for being a naturalist in the first place’ are indeed pragmatic ones in the sense that he intends them to be. What matters now is not that some practice works for human purposes, but that we treat that practice as if it worked. That it is the ‘scientific method’ and not ‘the holy book’ or ‘the holy leader’ that provides us with the dominant epistemic values today would be an observation about contingent social attitudes and not about the kind of skills with which the practice of giving natural scientific explanations provides human beings. More importantly, once we distinguish between (i) our interest in what a practice is doing for us and (ii) the normative standards with which the practice works, we can see that it is possible for us to share an interest in predicting the future and an attitude towards *that* interest with pre-Enlightenment folk, and simultaneously reject the specific standards that those folks employed in justifying their beliefs.

72 Of course, we never really take ourselves out of nature.

73 Robert Brandom, *Perspectives on Pragmatism: Classical, Recent, and Contemporary* (Harvard UP 2011) 5.

74 Once one recognises this, one can see that discursive intentionality is a kind of practical intentionality—a distinctive kind of practical intentionality, but practical nonetheless. ibid 9.

75 ibid 16. This framework is pragmatist in two ways: (i) the specific content of intentional states (such as belief) is understood as given by semantic (inferential) relations that involve the practical mastery of a language; and (ii) the use of that particular language is ultimately vindicated by the contribution that it makes to the satisfaction of some human need or interest. Such instrumental insight about meaning was summed up by John Dewey in a beautiful way: ‘Naturally, the pragmatist claims his theory to be true in the pragmatic sense of truth: it works, it clears up difficulties, removes obscurities, puts individuals into more experimental, less dogmatic, and less arbitrarily sceptical relations to life.’ John Dewey, *The Influence of Darwin on Philosophy, and Other Essays in Contemporary Thought* (Henry Holt and Company 1910) 164.

76 ‘Without a representationalist conception of the talk, however, the puzzle takes a very different form. It remains in the linguistic realm, a puzzle about a plurality of ways of talking, of forms of human linguistic behaviour. The challenge is now simply to explain in naturalistic terms how creatures like us come to talk in these various ways. This is a matter of explaining what role the different language games play in our lives—what differences there are between the functions of talk of value and the functions of talk of electrons, for example. This certainly requires plurality in the world, but of a familiar kind, in a familiar place. Nobody expects human behaviour to be anything other than highly complex. Without representationalism, the joints between topics remain joints between kinds of behaviour and don’t need to be mirrored in ontology of any other kind.’ Huw Price, ‘Naturalism without Representationalism’ in Huw Price and others (eds), *Expressivism, Pragmatism and Representationalism* (CUP 2013) 20.

77 ‘[E]xperimental jurisprudence can help assess questions about intuitions that are hard to address from the armchair.’ Kevin Tobia, ‘Experimental Jurisprudence’ (2022) 89 U Chi L Rev 743; This is also how experimental philosophy more generally has been understood. See Kornblith (n 3) 152.

78 Tobia has stressed the connection between experimental and ‘traditional’ jurisprudence, and thus also shown us the limitations of the former. See ibid 743.

79 Because this is only a methodological and not an ontological dualism, it poses no particular difficulty to a subject naturalism of the kind formulated in the last section.

80 A separate work is required to further explain the fact that, thus conceived, general jurisprudence presupposes a thesis about the unity of legal reality. In a very interesting piece, David Plunkett and Daniel Wodak have argued that we should take seriously the possibility that legal reality is disunified. According to Plunkett and Wodak, this disunity would explain why analytical jurisprudents, on one side, and legal realists and critical legal theorists, on the other, seem to be concerned with different things in putting forth their respective claims. See David Plunkett and Daniel Wodak, ‘The Disunity of Legal Reality’ (2022) 28 Legal Theory 235. Part of the beauty of the methodological pluralism for which I advocate is, however, precisely that it would make it possible for us to explain how scholars involved in such different enterprises may indeed all have one and the same object of study in mind.

81 In a very recent book, Julie Dickson has emphatically called attention to this complexity. The dual nature of law (as a social and normative phenomenon) imposes constrains on legal theory. See Julie Dickson, *Elucidating Law* (OUP 2022) 85. It is a curious fact that she does not take this principle to its actual methodological consequences: general jurisprudence should work from both an internal and an external perspective, the latter being best represented by the results of social scientific research and the conclusions obtained from critical perspectives on law.

82 A dualism that seeks to explain law from the perspective of the participants of the practice and from the perspective of an external observer; to explain the self-understanding of participants (that part of the self-consciousness of a community that is *legal*) and law as a causal phenomenon; to explain law from the perspective of the first person and from the perspective of the third person; or to formulate our explanations by reference to normative properties and also by reference to nomic or descriptive ones.

83 Technically, the latter two are already included in the former: ‘A consilient theory unifies and systematizes.’ Paul R Thagard, ‘The Best Explanation: Criteria for Theory Choice’ (1978) 75 The Journal of Philosophy 82.

84 ibid 82. Thagard adds: ‘Roughly, a theory is said to be consilient if it explains at least two classes of facts. Then one theory is more consilient than another if it explains more classes of facts than the other does. Intuitively, we show one theory to be more consilient than another by pointing to a class or classes of facts which it explains but which the other theory does not.’ See ibid 79.

85 This methodological dualism unifies classes of facts better because it explains why some of the properties that law can be said to have from the perspective of a non-participant follow from the fact that individuals understand themselves in a certain way as participants of the practice. That these are different classes of fact, however, is not an ontological proposition. The skills with which law provides us, and whose successful exercise and development require us to understand and explain how to use a particular kind of normative discourse, always belong to us natural creatures.

86 David Plunkett and Scott Shapiro, ‘Law, Morality, and Everything Else: General Jurisprudence as a Branch of Metanormative Inquiry’ (2017) 128 Ethics 47.

